# The effect of climate change and temperature extremes on *Aedes albopictus* populations: a regional case study for Italy

**DOI:** 10.1098/rsif.2024.0319

**Published:** 2024-11-06

**Authors:** Miguel Garrido Zornoza, Cyril Caminade, Adrian M. Tompkins

**Affiliations:** ^1^ The Niels Bohr Institute, University of Copenhagen, Blegdamsvej 17, Copenhagen 2100 Ø, Denmark; ^2^ Earth System Physics, Abdus Salam International Centre for Theoretical Physics (ICTP), Strada Costiera 11, Trieste, Italy

**Keywords:** *Aedes albopictus*, climate change, temperature extremes, regional modelling, vector-borne diseases, dynamical modelling

## Abstract

The Asian tiger mosquito, *Aedes albopictus*, has spread widely throughout Italy since its introduction, with significant public health implications. We examine how decadal temperature trends and sub-monthly heatwave events affect its climate-driven geographical distribution and temporal dynamics using a new regional-scale dynamical *Aedes* model. The model is calibrated using 12 years of ovitrap data for Emilia-Romagna and reproduces the vector seasonality and, to a lesser extent, its inter-annual variability. Simulated vector density hotspots overlap with densely populated areas in Rome, Milan, Naples, Foggia, Catania, Palermo, Lecce, Cagliari, Genoa, Turin and large urban centres in Emilia-Romagna. Lower risk is simulated over the Central Apennine mountains and the Alps. At decadal time scale, we simulate a lengthening of the active mosquito season by 0.5–3 weeks per decade, with the vector becoming homodynamic in southern Italy. Depending on the climatic setting, heatwaves can increase or reduce vector populations and, in some locations, can temporarily decrease mosquito populations. Such decreases can be followed by a population rebound and overshoot. Given the model’s skill in reproducing key spatio-temporal *Ae. albopictus* features, there is potential to develop an early warning system to inform control efforts at a national scale.

## 1. Introduction

*Aedes albopictus* (Skuse, 1894) (Diptera: Culicidae), most commonly known as the Asian tiger mosquito, is indigenous to tropical and subtropical regions of southeast Asia [1]. Even though it originates from forested areas, it is extremely well adapted to the urban environment [2], being able to use man-made artificial objects, such as tyres and gutters as breeding sites [3]. By increasing movement of goods, globalization has enabled this mosquito species to successfully invade many parts of the world [1], including temperate areas of Europe [4] and North America [5]. The colonization of Europe by the Asian tiger mosquito involved three independent introductions, very likely from used tyres in containers shipped from China. The first one occurred in Albania during the late 1970s, followed by introductions in northern and central Italy during the 1990s [6]. *Aedes albopictus* then rapidly spread from Albania and Italy to neighbouring European countries using motor vehicles and ships [7].

The establishment of the Asian tiger mosquito is of special public health concern due to its role as a competent vector of arboviruses such as dengue (DENV) [8], chikungunya (CHIKV) [9] and Zika virus (ZIKV) [10]. Autochthonous cases of dengue and chikungunya have been reported in southern France, Italy, Croatia and Spain over the past decade [11]. The first outbreak of chikungunya was reported in the province of Ravenna in Italy in 2007, with about 200 cases [12]. In 2023, about 80 dengue cases were reported in Lombardia and the Lazio region of Italy [13].

Similar to other ectothermic arthropods, *Ae. albopictus* proliferates at a set range of temperatures determined by the sensitivity of the gonotrophic cycle as well as survival rates during aquatic and aerial life stages. Thus, climate change in terms of the increase in both global surface temperature and changes in the occurrence of weather extremes, e.g. the frequency and intensity of heatwaves [14], not only has a significant direct impact on human health [15] but may also have it indirectly by affecting *Ae. albopictus* populations [16,17].

Past modelling studies, published in the early 2010s and based on environmental data, anticipated the spread of the Asian tiger mosquito in Europe [16,18,19], and have primarily focused on the long-term impact of climate on mosquito population dynamics. These studies underlined that recent climate change caused more favourable overwintering conditions, longer activity seasons, as well as a potential spread of this species to central-northern European countries. More recently, modelling studies have shown that *Ae. albopictus* could become homodynamic, namely able to breed all-year round, over southern Europe in the future [20]. Another recent global ecological niche study confirmed that *Ae. albopictus* could contribute to the emergence of chikungunya outbreaks and clusters of dengue autochthonous cases in southern France, Spain and Italy [21]. The Lazio region, which includes the metropolitan city of Rome and its international airport, with established *Ae. albopictus* populations, has already experienced autochthonous cases of dengue and chikungunya, and could potentially be at risk of yellow fever infections [22].

In contrast to longer-term climate change, relatively few studies have investigated the impact of short-term weather extremes, such as heatwaves, on *Ae. albopictus* population dynamics in a long time period. In previous work, a mechanistic mathematical model was used to show that heatwaves might be beneficial for mosquito development in the short term while having an overall detrimental impact [23]. Results are, however, strongly dependent on heatwave timing and intensity. Another study highlights that winter heatwaves favoured off-season survival of diapausing eggs [24].

In this study, we aim to further understand the impact of heatwaves in the context of a warming climate, investigating the effect of daily temperature on *Ae. albopictus* populations. We use a climate-sensitive mathematical vector model to simulate population dynamics at different mosquito life stages in Italy, a major hotspot for this invasive species [25]. Spatio-temporal ovitrap monitoring data are available over a 12-year time period for the Emilia-Romagna region, thus allowing a stringent validation and calibration of the model. The objectives of this study are twofold. First, following a thorough validation of the model, we aim to determine trends in seasonal activity and mosquito abundance hotspots in the vicinity of densely populated regions of Italy. Second, we conduct sensitivity experiments to tease out detrimental from beneficial effects of heatwaves on mosquito dynamics, providing a mechanistic interpretation based on the mosquito life cycle as well as the aggregated overall effect of these extreme events at a decadal time scale. Finally, we provide recommendations for public health stakeholders and discuss future perspectives of this work.

## 2. Methods

### 2.1. Model

We use the VECtor-borne disease community model of ICTP, TRIeste (VECTRI) model, which was originally developed for modelling the life cycle of *Anopheles gambiae* s.s. and associated *Plasmodium falciparum* malaria transmission [26–29]. The model explicitly resolves the mosquito life cycle, including the gonotrophic and larval cycles and has been progressively expanded to model additional mosquito species. From v. 1.11, it includes a parametrization suite for the Asian tiger mosquito, *Ae. albopictus*, including a new temperature-dependent survival scheme [30]. The model version used here is v. 1.11.3.

The key model inputs are 2 m air temperature, which impacts the gonotrophic and larval growth rates, as well as larval and vector mortality, and precipitation, which provides breeding sites. An important parameter in the model is the water coverage of each model grid cell, which can serve as potential breeding sites, given as a fraction w(λ,ϕ,t)∈[0,1], since it controls the instantaneous carrying capacity of larvae biomass. The parameter represents a subset of all water coverage since large bodies of water are unsuitable for mosquito breeding. This potential breeding site coverage, or fraction of potential breeding sites, is built using a variety of climatic, hydrological and human-related features as proxies for the presence of potential breeding sites, constituting their aggregated value. Concretely, from v. 1.10 of VECTRI, this fraction is composed of three categories: the presence of sites that can occur along the borders of permanent features such as rivers or natural lakes ($w_{perm}$), urban reservoirs such as water storage containers and plant pot drip trays ($w_{urbn}$) and precipitation-fed temporary ponds ($w_{pond}$). The $w_{\text{pond}}$ category is the only one that evolves dynamically in time in response to rainfall [31,32]. In rural environments, this category refers to ephemeral pools while in urban environments it also includes rain-fed sites such as roadside ditches and poorly draining gutters. In contrast, $w_{urbn}$ and $w_{\text{perm}}$ are time-invariant. The first simply relates the availability of urban breeding sites proportionally to the logarithm of human population density [33,34], similar to other models that use human presence to estimate part of the local carrying capacity [35,36]. The permanent fraction, $w_{perm}$, is derived from aggregating water–land border pixels using metre-scale resolution Sentinel-derived land-cover maps aggregated to 5 km tiles, but is not used in this study. The model does not account for land surface cover, nor does it represent the transport of vectors over long distances, e.g. by motor vehicles, currently assuming a small seed vector population in each location when initializing from an artificial initial state. We note that the carrying capacity related to breeding site availability is one of the greatest sources of uncertainty in the model, as it is very difficult to evaluate from observations.

For a given mosquito vector parametrized in VECTRI, the total availability of breeding sites, w, is the sum of each water body class i, weighted by a species-specific usage coefficient, $r_{i}$, which represents the relative breeding habitat preferences of this vector:


(2.1) $$w=r_{pond}\cdot w_{pond}+r_{urbn}\cdot w_{urbn}+r_{perm}\cdot w_{perm}.$$

Thus, *An. gambiae* s.s. that is primarily found in rural settings [37], would have a high coefficient of $r_{\text{pond}}$, close to unity, while $r_{\text{urbn}}$ is close to zero. *An. funestus* would instead have a larger value for $r_{\text{perm}}$ [38–40], while urban-adapted species such as *An. stephensi* [41,42] or *Ae. albopictus* [43,44] should have larger $r_{\text{urbn}}$ values but also a non-zero pond usage fraction, while $r_{\text{perm}}$ is set to zero. The $r_{i}$ usage coefficients are highly uncertain and subject to the calibration process outlined in §2.3.

### 2.2. Input data

The model is driven by daily rainfall (mm) and 2 m air temperature, $T_{2m}$ (°C). As climate data input, we used the daily E-OBS dataset version 28.0e [45] for the period 1980–2023, with an approximately 0.1° × 0.1° spatial resolution. We used population density estimates from the Gridded Population of the World GPwv4 project [46] to calculate $w_{\text{urbn}}$. Population data (per km^2^) was interpolated to the climate data grid resolution using a conservative interpolation method with the CDO software v. 2.30 [47].

**Figure 1.**
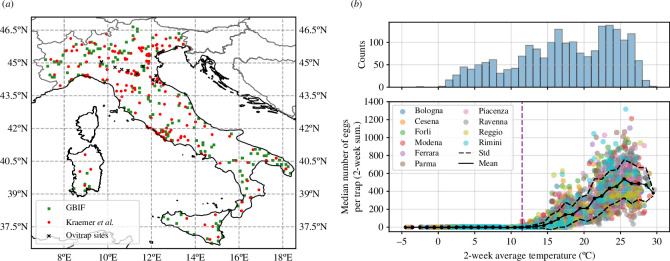
(*a*) The Italian sites used for the model calibration (black crosses) and map of reported observations of *Ae. albopictus* based on data from [54] (red circles) and [55] (green squares). (*b*) Egg abundance (two-weekly totals per trap) as a function of the bi-weekly average $T_{\text{2m}}$ for the 10 Italian cities. The mean (dotted-solid) and mean ±1 standard deviation (dashed) are shown using 1°C bins. On top, we show the associated temperature histogram.

VECTRI has a set of mosquito-related constant parameters which can be constrained by field and laboratory observations but are nevertheless uncertain. Employing a particle filter genetic algorithm (GA) methodology [48–50], we performed a constrained optimization and calibrated these parameters against temporal egg data, which are monitored by a network of ovitraps deployed in 10 Italian cities of the Emilia-Romagna region [51–53] (black crosses in figure 1*a*). This extensive surveillance network was set up shortly after the 2007 Chikungunya outbreak caused by *Ae. albopictus* [56–58]. We used median ovitrap data for the cities of Bologna, Cesena, Forli, Modena, Ferrara, Parma, Piacenza, Ravenna, Reggio and Rimini, from which the first half were used in the calibration and the rest were left as independent data for a posterior validation of the model. These data are recorded bi-weekly and we used the period 2010–2022 for calibration. In this study, we adjust $r_{\text{urbn}}$, given the preferential affinity of *Ae. albopictus* for urban sites [2], and $r_{\text{pond}}$. The remaining adjusted parameters and further details on the calibration process are provided in electronic supplementary material, S1.

Once calibrated against ovitrap data, VECTRI was validated against this temporal data, including all cities, as well as against spatial information on the presence of *Ae. albopictus* in the Italian peninsula and Sardinia (see §2.4). Spatial occurrence data were derived from two sources: the study by Kraemer *et al.* [54] and the Global Biodiversity Information Facility (GBIF) [55] (figure 1*a*).

### 2.4. Validation metrics

To validate our model temporally, we calculated Pearson correlation coefficients between simulated and observed egg abundances, e(t), for the 10 Italian sites, as follows.

#### 2.4.1. Seasonality

The first correlation coefficient, $r_{site,all}^{2}$, calculates the correlation for all bi-weekly time points over the study period (2010–2022) where data are present and sufficiently continuous, i.e. there are no large missing data gaps throughout June–July–August (JJA). This metric is frequently employed in model validation to quantify the model’s ability to accurately reproduce observed trends [30,59,60]. Since the vector population density signal is strongly seasonal these coefficients assess how well the model captures the mosquito seasonality.

#### 2.4.2. Inter-annual variability

Complementary to the seasonality metric, we also calculate Pearson correlation coefficients using standardized annual mean egg abundances in order to remove seasonality and focus on the ability of the model to represent inter-annual variability. Given that data were missing for Parma, Piacenza, Reggio and Rimini, the calculation is made for the multi-year time domain 2014–2021 for individual sites and referred to as $r_{site,y}^{2}$.

Assessing inter-annual variability is extremely challenging, however, as non-climatic factors such as vector control measures, micro-climatic features as well as large uncertainties associated with ovitrap data, will cause site-to-site differences that may considerably exceed those driven by climate data, available at coarser spatial scales [53,61]. One way to try to account for such differences is to consider the spatial scales of climate temperature anomalies, since inter-annual variability and decadal trends of temperature will be relatively uniform across the scale of the ovitraps sites, i.e. the inter-trap distances are relatively small compared with the spatial scale of temperature anomalies. This assumption holds for precipitation, but to a lesser degree, since precipitation can be more spatially heterogeneous with respect to temperature on inter-annual time scales (see electronic supplementary material, S2.1). Thus, by constructing the correlation between each model site and the ensemble mean across all Italian observation sites, $r_{ens,y}^{2}$, we aim to isolate the climate-driven signal from other factors and data errors that operate on the sub-regional scale. These statistics are calculated as well over the period 2014–2021.

A perfect match between the observed and simulated vector density would mean $r^{2}∼1$. A lower value of either indicates flaws in different aspects of the simulated signal, as described above.

#### 2.4.3. Spatial validation

To measure the ability of our model to discriminate regions where *Ae. albopictus* is present against regions where it is absent we constructed receiver operating characteristic (ROC) curves [62] using the aforementioned spatial occurrence data. This spatial validation metric, used extensively in species’ distribution modelling [63], required the conversion of our model outputs into a binary format, i.e. into presence–absence data. This synthetic presence–absence data were then compared against actual occurrence data to quantify their overlap (see electronic supplementary material, S2.2). Specifically, to map our continuous data into 0s and 1s (stating the absence or presence of the mosquito in a particular site, respectively), we used the vector density variable, averaged over the simulated period, as a classifier. If a given threshold value in the vector’s density (which was changed parametrically to construct the curve) was (not) exceeded by the modelled vector density, then the mosquito was considered to be present (absent) in that site. Finally, if this agreed with the observations, we then marked it as a true positive (negative), otherwise we considered the model had failed to properly predict the absence (presence) of the mosquito in that site, i.e. the test was considered a false positive (negative). From this, we report the area under the curve (AUC), i.e. the integral of the ROC curve. As an integrated quantity, the AUC is a threshold-free indicator of the general ability of the model to weight areas particularly suited for a given species. A random predictor model has an AUC of 0.5, a good model lies between approximately 0.6 and 0.8, and an excellent predictive model is above 0.9.

### 2.5. Experimental set-up

Once calibrated and validated, we analysed two simulations. First, a control simulation, termed *cntl* hereafter, with unmodified observed daily values for temperature and rainfall. Second, a counterfactual simulation, where the temperature series has been modified to remove warm events, and thus does not contain their contribution to the simulated mosquito’s population density. Heatwaves are events with extreme temperature values. There are, however, many ways to define an extreme [64,65], most revolving around the choice of threshold. Some studies work with fixed thresholds, such as 25°C [66,67], trending thresholds, to correct for the non-stationary baseline provoked by global warming, or thresholds based on the local distribution of the variable [65,68], such as percentile-based thresholds. In this study, we used the boreal summer JJA 90th percentile for the period 1980–2023 as a simple heatwave threshold in each grid cell. Threshold values are therefore local and, by construction, 10% of all events are considered to be extreme. In practice, temperature values exceeding the 90th percentile were clipped to this value, i.e. if $T_{2m}>T_{90th}$ then we set $T_{\text{2m}}=T_{90th}$. We shall denote this second simulation as *clipped*. Our choice of threshold is based on the interest to capture the effect of heatwaves relative to local conditions in a changing climate while keeping the physiological characteristics of the mosquito constant.

## 3. Results

### 3.1. Ovitrap data

The relationship between observed bi-weekly egg abundance and two-week average temperature for cities monitored in the Emilia-Romagna region is depicted in figure 1*b*. Even though some eggs were found below 10°C, most eggs were trapped at temperatures exceeding approximately 11°C. The average across all sites surpasses one egg per trap in the 11–12°C bin, denoted by a vertical dashed line. These temperature thresholds are consistent with early risk modelling assumptions for this species in the UK [69] and Japan [70]. The peak egg densities appear to occur between 25°C and 26°C after which the egg density starts to decline. In the period and location where eggs were sampled, average bi-weekly temperature never exceeded approximately 30°C. Egg abundance values for the 10 Italian cities are relatively homogeneous, with the largest abundance values being recorded in Rimini.

### 3.2. Model validation

#### 3.2.1. Seasonality

**Figure 2.**
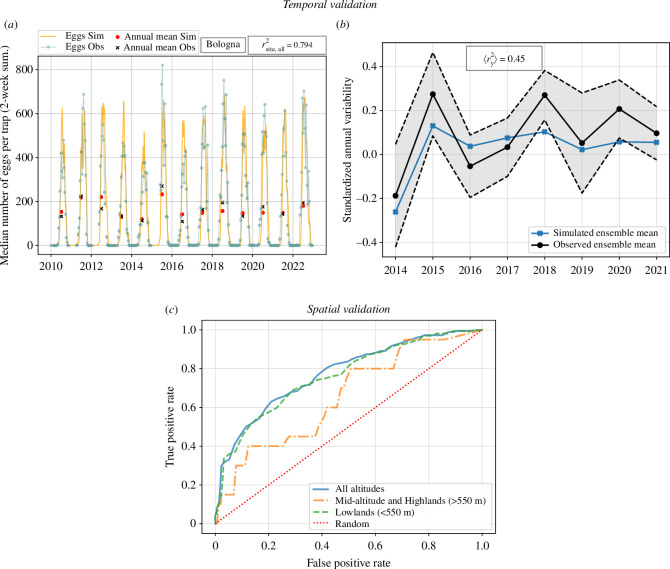
(*a*) Example of the temporal calibration in the Bologna site using the GA. Observed egg data are shown against rescaled model output (see electronic supplementary material, S1) as well as their respective annual means. (*b*) Standardized ensemble annual means of the observed and simulated (*cntl*) egg densities in the 10 Italian sites. (*c*) Example ROC curves for the whole Italian domain, the mid-altitude and highland regions (greater than 550 m) and the lowland areas (less than 550 m) using baseline data from [54].

The seasonality of the observed egg population is well-captured at the ovitrap sites (Bologna in figure 2*a* and electronic supplementary material, S3). Importantly, our model captures the start and end of the observed egg activity season for all studied sites with a small delay in the onset phase relative to the data. In table 1 (left, first column), we report the seasonal performance of the model for the 10 different Italian sites. Most correlation coefficients exceed 0.8 (except Ravenna), and all are significant at the 99% confidence interval. We emphasize that the calibration technique only allows the specified constants of the mathematical dynamical model to be adjusted within the bounds of their assessed uncertainty [48], the prior, and in this respect contrasts with a free parameter search or the free fitting of a statistical model such as commonly used generalized linear models. In this sense, the constrained optimization approach resembles a Bayesian inference method, such as the one used in a similar study [35], from the use of prior and bounded information in the search for an optimal, yet realistic, solution. The fact that the model is able to simulate the seasonal evolution is only possible if the underlying equations that describe the larvae–adult life cycles are reasonable approximations of the biological system under scope.

**Table 1.** Left. Temporal validation of the VECTRI model against egg data from the Italian sites. We report $r_{site,all}^{2}$, $r_{ens,y}^{2}$ and $r_{site,y}^{2}$ for each city. We mark significant results at the 90% (*), 95% (**) and 99% (***) confidence intervals. Right. Spatial validation of the model against two occurrence databases of observed *Ae. albopictus*.

**T**emporal				Spatial	
Location	$r_{site,all}^{2}$	$r_{ens,y}^{2}$	$r_{site,y}^{2}$	AUC	Height
Bologna	0.79***	0.50**	0.84***	[54]	
Cesena	0.69***	0.61**	0.12	0.77	All
Ferrara	0.72***	0.70***	0.48*	0.76	< 550 m
Forli	0.71***	0.06	0.53**	0.66	> 550 m
Modena	0.68***	0.22	0.14	[55]	
Parma	0.78***	0.49*	0.63**	0.77	All
Piacenza	0.81***	0.50*	0.77***	0.75	< 550 m
Ravenna	0.63***	0.47*	0.67**	0.84	> 550 m
Reggio	0.72***	0.43*	0.51**	[54] & [55]	
Rimini	0.80***	0.50**	0.11	0.69	All
				0.66	< 550 m
				0.72	> 550 m

#### 3.2.2. Inter-annual variability

$r_{ens,y}^{2}$ correlation coefficients at inter-annual time scale are moderate, and mostly significant except for Forli and Modena, where the model clearly disagrees with the observed variability (table 1 left, second column). Most points of the simulated ensemble mean, however, lie within 1σ of the observed ensemble mean (figure 2*b*), indicating that part of the climate-driven variability signal is captured by the model. $r_{site,y}^{2}$ correlation coefficients for Cesena and Ferrara are lower than their ensemble equivalent, due to some years having opposite trends between on-site simulated and observed signals (2017 and 2020 for Cesena, and 2017 for Ferrara). Ravenna, Reggio, Piacenza, Forli and Parma show an improvement that misrepresents the quality of the simulated signal, given that sporadic missing data in these locations during the peak vector activity weights the metric towards a seasonality estimate (see electronic supplementary material, figure S3, in electronic supplementary material, S3). Six out of 10 site-to-site correlation coefficients ($r_{site,y}^{2}$) are significant at the 95% confidence level, denoting the model’s capability in reproducing low–high egg abundance years per city. However, these correlation values should be considered carefully, given the small sample size (8y) and the amount of missing data.

#### 3.2.3. Spatial validation

The spatial validation is performed using different subdomains, each defined for different altitude ranges (figure 2*c*). In table 1 (right), we report the area under the ROC curve, AUC, for the different altitude strata and datasets. Most AUC exceed 0.7, and there are differences depending on the observed occurrence data that were used as a baseline. Notably, AUC exceed 0.75 when using the latest, most up-to-date, occurrence database from the Global Biodiversity Information Facility as a baseline. Consequently, the model is able to reproduce the geographical extent of the vector to a good degree, especially accounting for the limitations of such databases and the fact that the vector is still in a phase of expansion and may still not have invaded all possible climatically suitable niches within the country, as reflected by its recent spread to higher altitude regions of Italy [71].

### 3.3. Average risk: 1980–2023

**Figure 3.**
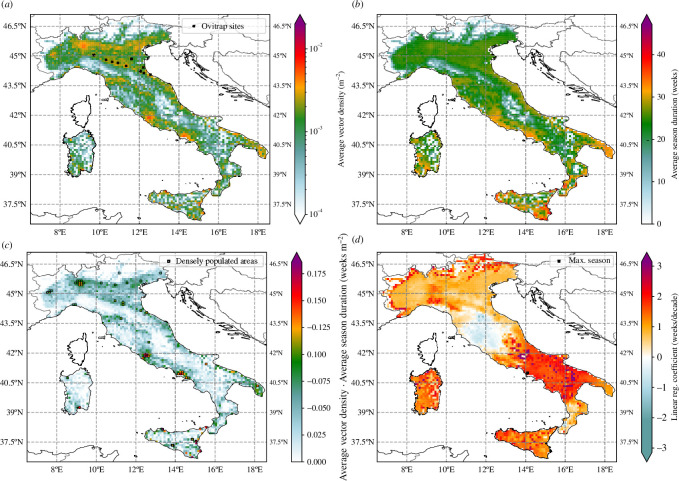
(*a*) Mean vector density for the 1980–2023 period. The Italian sites used for the model calibration are depicted again with black crosses. (*b*) Average season duration (weeks), defined as the number of days in the year where the vector density is above $1.5\cdot 10^{-4}$ m^−2^. (*c*) As a plausible metric to describe the average level of risk, we here show the product of (*a) and (b*). (*d*) Linear regression coefficients of simulated activity season duration (weeks/decade) across Italy. In black, we have masked regions where the season has already reached 365 days at least 1 year throughout the simulated period.

We examine the averaged vector density for the *cntl* simulation (figure 3*a*), recalling that the model assumes that *Ae. albopictus* has been introduced at all locations and thus simulates population dynamics solely based on the local climatic conditions.

Densely populated urban areas such as Milan, Rome and Naples are simulated to be the main *Ae. albopictus* hotspots. The Po Valley, the Firenze area and the Apulia region (SE) also show large simulated density values, whereas highland areas such as the Central Apennine mountains show low density values. There is a pronounced north–south gradient in the simulated length of the vector activity season (figure 3*b*). Longest activity seasons (approx. 30–40 weeks) are simulated over southern coastal areas (Puglia, Basilicata and Calabria), the Lazio region, most of Sicily and parts of Sardinia. Northern regions including the Po Valley are simulated to experience shorter activity seasons (approx. 20–30 weeks). Since the average vector density in the Po Valley is, however, high this indicates a shorter but therefore more pronounced activity, as compared with southern regions that might have a longer season with lower vector population density values.

We provide a metric assessing the integrated risk of mosquito density and the length of its activity season in figure 3*c*, where we overlaid regions with human population densities above 1500 km^−2^, following the EU criterion for urban centres [72]. This map highlights regions where high simulated risk values coincide with densely populated areas. The largest simulated risk values coincide with population hotspots in Rome, Naples, Foggia, Catania, Palermo, Lecce and Cagliari. To a lesser extent, Milan, Genoa, Turin and large urban centres in the Emilia-Romagna region (Bologna, Modena and Ravenna) are also concomitant with high-risk values.

### 3.4. Decadal trends

We observe a linear trend in the mosquito season length, defined as the period where the vector density is higher than a small threshold value ($1.5\cdot 10^{-4}$ m^−2^; see electronic supplementary material, figure S4 for further details about threshold selection). In figure 3*d*, we show the heatmap of linear regression coefficients (slopes) across Italy. Most of the Italian peninsula and Sardinia experience an increase in the season length, with the exception of lower Tuscany and upper Lazio regions. The largest increasing trend is shown over the southernmost regions, with a lengthening of the activity season that ranges between two and three weeks per decade over the study period. According to the model, climatic factors can be suitable for the vector to remain active all-year round (homodynamic activity) over a few southern coastal areas (depicted by black squares in figure 3*d*).

### 3.5. Impact of short-term heatwave events

#### 3.5.1. Mean seasonal effect

By comparing *cntl* and *clipped* simulations, we can measure the integrated effect of warm temperature events across Italy. We see that, on average, heatwaves are beneficial to the vector and result in a net increase of the mosquito population (see electronic supplementary material, figure S5). The increase is nonlinear in time with the mean impact of heatwaves being larger in the 2010s with respect to the earlier period. This is the result of having a fixed 90th percentile threshold over the studied period, which is thus exceeded more frequently and by further in later years due to global warming. The spatial distribution of temperature-driven effects on vector density populations can be quantified by calculating the temporal covariance of the temperature difference and the rate of change of the egg density difference, between *cntl* and *clipped*, i.e.


(3.1) $$c(\lambda ,\phi )=cov\left[\Delta T_{2m}(\lambda ,\phi ,t)\cdot \frac{d(\Delta e(\lambda ,\phi ,t))}{dt}\right]\;.$$

**Figure 4.**
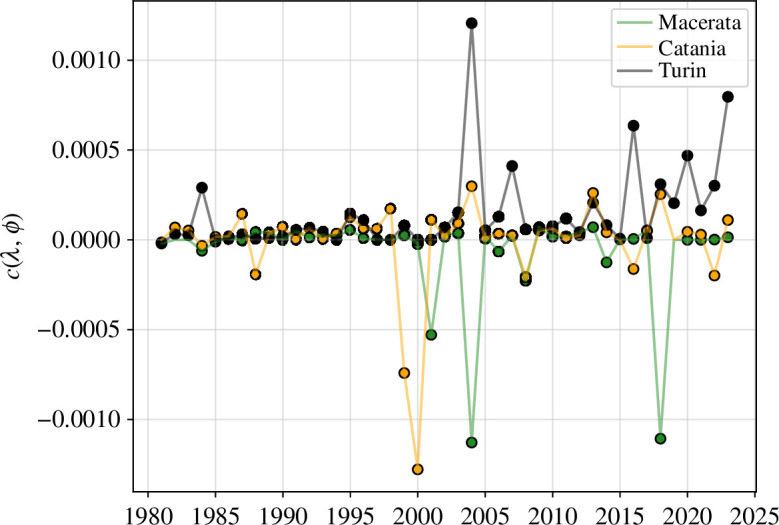
Temporal distribution of temperature-driven effects on mosquito populations in three Italian cities: Macerata (E), Turin (NW) and Catania (SE).

**Figure 5.**
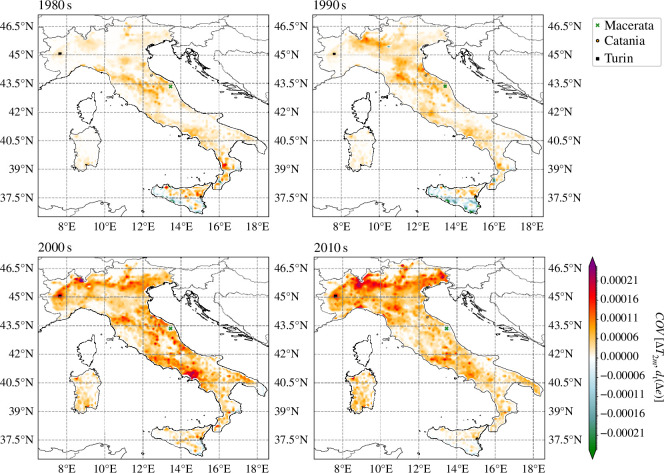
Covariance of the temperature difference and the rate of change in the egg density difference between the *cntl* and the *clipped* simulations for the 1980s, 1990s, 2000s and 2010s. Positive values mean that the increased temperature conditions, which we define as heatwaves, translate into an increase in simulated egg density, whereas negative values indicate a detrimental effect of higher temperatures. Here, we show the integrated effect per decade. We mark the location of the cities shown in the previous figure.

Here, $\Delta T_{2m}(\lambda ,\phi ,t)\equiv T_{2m}(\lambda ,\phi ,t)-T_{2m}^{90th}(\lambda ,\phi ,t)$ and $\Delta e(\lambda ,\phi ,t)\equiv e(\lambda ,\phi ,t)-e^{90th}(\lambda ,\phi ,t)$, with 90th denoting the *clipped* experiment. We chose to use the egg density in the covariance calculation since this variable shows a faster response to temperature changes than the vector density, which presents a small delay with temperatures due to inherent biological lags. Importantly, if the rate of change was calculated on the temperature difference instead of on the egg difference our metric would misrepresent the effect of increased temperatures, namely the first term could then be negative (positive) while the actual temperature difference, and thus the perturbation, was positive (negative) and the second term could be positive (negative) even though the last increased temperature had a detrimental (beneficial) effect on the egg population. An example case for three cities in distinct regions shows how temperature-driven effects are temporally distributed and its effect can vary widely across the Italian peninsula (figure 4). For Turin, covariance values are mostly positive over the study period, while they are mostly negative for Macerata (figure 4). For Catania, these can either be positive or negative depending on the year. Since we have already observed a long-term trend in the length of the mosquito activity season, we split the covariance calculation into decades 1980s, 1990s, 2000s and 2010s (figure 5). There are discernible spatial heterogeneities, which are accentuated in time. Despite representing a suitable habitat for *Ae. albopictus*, southern coastal regions, especially in Sicily, include areas where warm events increase the net mortality and thereby have a net detrimental effect on the mosquito population. There is, however, a clear tendency for these events to be beneficial elsewhere. Particularly, parts of the Po Valley and northern lowland regions, central valley areas in Trentino and the Rome–Naples coastal urban areas have experienced a clear beneficial effect of warm events on mosquito population at decadal time scales.

#### 3.5.2. Sub-monthly dynamics

The long-term mean responses mask short-term impacts that can be positive or negative for the mosquito, as suggested by figure 4. In order to demonstrate this, we have identified three types of short-term responses that we illustrate with three case studies.

*Case 1. Consistently beneficial events*

**Figure 6.**
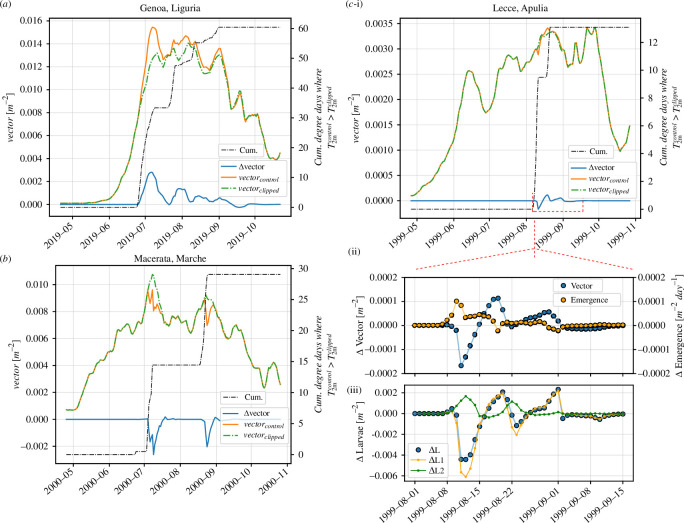
(*a*) Consistently beneficial warm events. We here show the *cntl* and *clipped* vector densities in Genoa during the summer of 2019, their difference, Δvector, and the cumulative number of degree days when the *cntl* temperature has exceeded the *clipped* one. The former indicates the time, magnitude and duration of warm events. (*b*) Consistently detrimental warm events. (*c*) Temporarily detrimental warm event. (i) Example case in Lecce in 1999. (ii) Zoomed-in vector density throughout the heatwave accompanied by the emergence rate, i.e. the rate at which larvae transition to the vector state. As before, population densities are the difference between *cntl* and *clipped*. (iii) Larvae dynamics for the total population, ΔL, young larvae $\Delta L_{1}$ (aggregate of the first bins of VECTRI’s bin-resolved larval age structure) and old larvae $\Delta L_{2}$ (aggregate of the remaining bins).

In this example, the warm events remain within a ‘beneficial’ temperature suitability range. Namely, the temperature-induced vector mortality is secondary compared with temperature-induced increases in the larval and adult growth rates, and thus mosquito populations tend to increase incrementally throughout the whole duration of the heatwave. In figure 6*a*, we show an example for Genoa where the aforementioned criterion is true for the whole activity season. At the start of the season, vector density values for both *cntl* and *clipped* experiments remain identical. However, in late June 2019, temperatures start rising above the 90th JJA percentile, as depicted by the cumulative number of degree days (black line), and population densities start to diverge between *cntl* and *clipped* simulations. This beneficial effect, namely that the vector density in the *cntl* simulation is systematically larger than the *clipped* analogue, remains the same until September 2019. In other words, temperatures did not reach values large enough to have a net detrimental effect on the simulated vector density in Genoa in 2019.

*Case 2. Consistently detrimental events*

Analogously to the previous case we find situations where extreme temperatures have a detrimental effect to vector populations. Such is the case in Macerata in 2000 (figure 6*b*), where temperatures exceeded the 90th percentile twice, in early July and late August, both resulting in a simulated decrease in mosquito populations. This example case study of Macerata, where the two major heat events are separated by a long period, enables us to ascertain a lag between the climate and *Ae. albopictus* population, observed in a different *Ae. albopictus* modelling study focused on subtropical regions of China (Guangzhou) [23]. The extreme heat starts to have an immediate impact on vector populations due to the increase in mortality, but the effect of the heatwave continues to be felt for the first 8 days after the event termination due to the recovery associated with the vector life cycle (figure 6*b*). The recovery time scale will itself be dependent on temperature, being higher at cooler temperatures given the decreased larval and gonotrophic development rates.

*Case 3. Temporarily detrimental*

In this category, warm events occasionally lead to periods of lower vector density due to the detrimental impact of extreme temperatures on mosquito survival. These periods are, however, followed by a population density rebound in *cntl*, which, in some cases, exceeds the *clipped* equivalent. In figure 6*c*(i), we show an example for Lecce, in the region of Apulia. During the heatwave that occurred in August 1999, vector and larval densities are initially reduced in *cntl* with respect to *clipped* (figure 6*c*(ii,iii)). This is especially true for larvae in their early development stage (figure 6*c*(iii)), where increased temperatures act to decrease young larval density while increasing the older larval density and the emergence rate (the rate at which larvae transition to adult vectors). Such increases are concomitant with a higher survival probability due to lower overcrowding effects and indicate a shift of the distribution towards further developed larvae. A couple of days later, this effect leads to the simulated overshoot in vector and larval populations. The cause of these transient dynamics might therefore be related to the bin-resolved age structure of larval development in the VECTRI model which is investigated further below.

Overall, such differences occur during periods when the *cntl* 2 m air temperature, $T_{2m}$, is above the *clipped* analogue. When both temperatures are again identical, simulated vector population densities of *cntl* and *clipped* tend towards the same value, with some transient relaxation caused by the finite memory of the model. Further examples of detrimental/beneficial cases are shown in electronic supplementary material, S5.

### 3.6. Conceptual models

In order to better understand the underlying mechanisms driving the observed system response to warm events (case 3), we study the dynamics of two simplified models of the ecology of the vector, eggs and larvae. The aim is to identify the key features that provoke the transient behaviour observed in the VECTRI model. The most basic representation that explicitly resolves vector (V) egg (E) and larval (L) densities, is a three-state model of the type


(3.2) $$\begin{array}{rl} \frac{\text{d}V}{\text{d}t} & =\alpha_{\text{L}}\cdot L(t)-\delta_{\text{V}}(t)\cdot V(t) \end{array},$$

(3.3) $$\begin{array}{rl} \frac{\text{d}E}{\text{d}t} & =N_{\text{eggs}}\cdot \alpha_{\text{V}}\cdot V(t)-\alpha_{\text{E}}\cdot E(t)-\delta_{E}(t)\cdot E(t) \end{array},$$

(3.4) $$\begin{array}{rl} \frac{\text{d}L}{\text{d}t} & =\alpha_{\text{E}}\cdot E(t)\cdot \left(1-\frac{L(t)}{K}\right)-\alpha_{\text{L}}\cdot L(t)-\delta_{\text{L}}(t)\cdot L(t)\;, \end{array}$$

with $\alpha_{i}$ being the transition (larval and gonotrophical cycle development) rates, *N*_eggs_ the average number of laid eggs per batch, K the system’s carrying capacity and $\delta_{i}(t)$ a time-dependent mortality rate. The time dependence in the former is incorporated with the aim to model a transient increase, mimicking the effect of a detrimental warm event, on vector, larvae and egg mortality. As in VECTRI, larvae are here modelled to grow logistically up to a certain carrying capacity, K, specific to the environmental context. In VECTRI, the transition/growth and mortality rates are a function of temperature, given by relationships derived in laboratory experiments, while here are modelled to be constant. Furthermore, since the observed system response in the climate-aware model is not specific to a particular location or year we can safely assume transition rates are not fundamental and thus set them to unity, i.e. $\alpha_{i}=1\;\;\forall i\in [V,E,L]$. Time is therefore expressed in normalized generational units.

**Figure 7.**
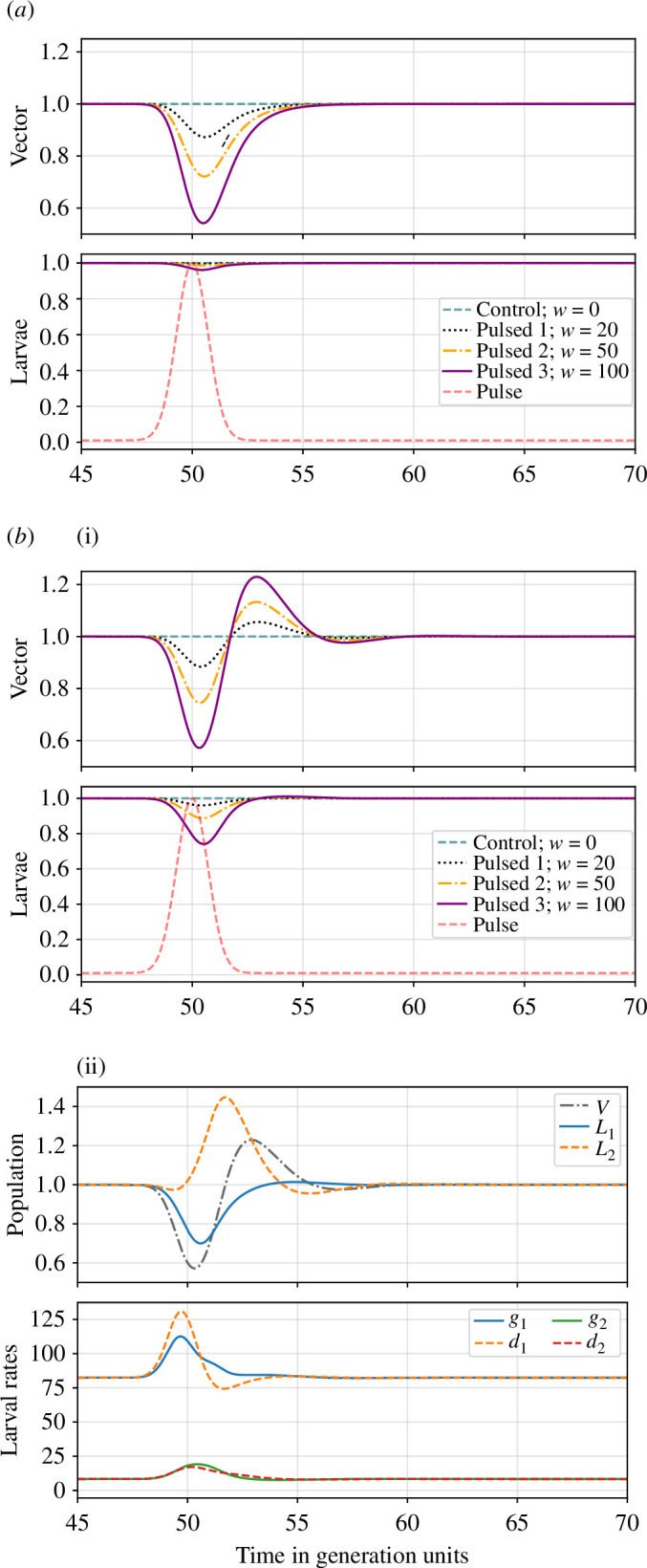
(*a*) Population dynamics of the ‘logistic’ model normalized by the respective steady-state values. In red/dashed, we show the normalized pulse. (*b*) (i) Population dynamics of the ‘age-structured’ model and (ii) the corresponding larvae dynamics along with their growth, $g_{i}$, and decay, $d_{i}$, rates for both young, $L_{1}$, and older, $L_{2}$, larvae for the case w=100.


We study the dynamics of our conceptual model against a transient increase in the decay rate, modelled as


(3.5) $$\delta_{i}(t)=\delta_{0}\cdot \left(1+w\cdot e^{\frac{-(t-t_{0}{)}^{2}}{\tau }}\right)+\delta_{i}\;,$$

i.e. as a Gaussian-like transient pulse centred at $t_{0}$ and with a spread of approximately τ. The second term in the right-hand side of the decay rate is let to be specific to the state (i=V, E or L). The magnitude of the pulse is controlled by the parameter w. The response of the conceptual model against this pulse is shown in figure 7*a*. Logistic growth does not suffice to qualitatively describe the observed behaviour. We thus expand the model to describe the age structure in larval populations, introducing two larval (L) stages: $L_{1}$ and $L_{2}$, that can be considered as an idealized analogue of first/second and third/fourth instar populations, respectively,


(3.6) $$\begin{array}{rl} \frac{\text{d}V}{\text{d}t} & =L_{2}(t)-\delta_{\text{V}}(t)\cdot V(t) \end{array}$$

(3.7) $$\begin{array}{rl} \frac{\text{d}E}{\text{d}t} & =N_{\text{eggs}}\cdot V(t)-E(t)-\delta_{\text{E}}(t)\cdot E(t) \end{array},$$

(3.8) $$\begin{array}{rl} \frac{\text{d}L_{1}}{\text{d}t} & =E(t)\cdot \left(1-\frac{L_{1}(t)+\alpha \cdot L_{2}(t)}{K}\right)-L_{1}(t)-\delta_{\text{L}}(t)\cdot L_{1}(t) \end{array},$$

(3.9) $$\begin{array}{rl} \frac{\text{d}L_{2}}{\text{d}t} & =L_{1}(t)\cdot \left(1-\frac{L_{1}(t)+\alpha \cdot L_{2}(t)}{K}\right)-L_{2}(t)-\delta_{\text{L}}(t)\cdot L_{2}(t). \end{array}$$

A key element of this model is the two larval stages sharing the available resources. Since older larvae tend to have a higher biomass and energy requirements, their contribution to the total carrying capacity should in principle be weighted by a factor approximately $\alpha \cdot L_{2}$. However, without altering the model behaviour, for simplicity we take α=1.

The dynamics of this age-structured model are shown in figure 7*b*(i). The bin-resolved age structure is a fundamental driver for the observed rebound, overshoot and relaxation dynamics. In figure 7*b*(ii), we show the larval density split in $L_{1}$ and $L_{2}$: upon the passing of the ‘heatwave’ pulse, younger larvae, whose steady-state population densities are higher, undergo a steep decrease, driven by the larger decay rate, $d_{1}$ (negative contribution in equation 3.8), as compared with the growth term, $g_{1}$ (positive contribution in the same equation). This is not the case for older larvae, $L_{2}$, whose decay and growth rates (analogously determined from equation 3.9) remain of the same magnitude, with the growth rate initially being slightly higher. This is caused by the respective decay and growth rates being proportional to distinct population densities, i.e. to $L_{1}$ and $L_{2}$ in equation (3.9). The shared carrying capacity acts now as a boost for older larvae, which find an empty niche to grow, increasing above their steady-state point and leading to the subsequent overshoot in vector population. The system parameters can be found in table 2.

**Table 2.** System parameters used in the logistic and age-structured models. The remaining parameters shown in the equations are provided in the main text.

K	$\delta_{0}$	$N_{e\,g\,g\,s}$	τ	$t_{0}$	$\delta_{V}$	$\delta_{E}$	$\delta_{L}$
$10^{2}$	0.01	100	1	50	1	0	0

This simplified model thus highlights a weakness in the dynamical VECTRI model. In reality, environmental resource limitations are mitigated for late-stage larvae through cannibalism of early stages [73], which can have a net benefit for larvae numbers reaching emergence [74,75]. This would act to smooth the impact of heatwaves on larvae numbers and could indicate that the rebound effect produced in some settings is exaggerated in the VECTRI model simulations.

## 4. Discussion

We have modelled the population dynamics of the Asian tiger mosquito, *Ae. albopictus*, an invasive species which is currently a threat to public health in Europe given its competence to transmit arboviruses. The climate-sensitive VECTRI model has been calibrated and validated against ovitrap field data, successfully reproduces the seasonal cycle and, to a lesser extent, the year-to-year variability in observed vector population. Importantly, our model accurately simulates the start and end of the mosquito activity season for the 10 Italian cities located in the Emilia-Romagna region. Spatially, the model captures the observed distribution of *Ae. albopictus* in Italy, with AUC values above 0.7. Our findings underline that simulated mosquito abundance hotspots coincide with densely populated centres in Rome, Naples, Foggia, Catania, Palermo, Cagliari, Lecce, Milan, Genoa, Turin and in most large cities of the Emilia-Romagna region.

Regarding global warming trends, we show a lengthening in the seasonal activity of *Ae. albopictus* in Italy which is more pronounced over southern regions, and can reach about three extra weeks per decade. Furthermore, we demonstrate that heatwave summer conditions can have both beneficial and detrimental impacts on simulated mosquito densities depending on the location and year under focus. Beneficial impacts tend to occur when temperatures increase larvae growth rates and decrease the gonotrophic cycle time, which dominate decreases in vector survival. On the other hand, detrimental effects occur when temperatures tend to increase larval mortality in the model to such an extent that they overcome the increased growth rates. In some cases, such effects can be followed by a subsequent rebound related to the system’s carrying capacity and biological delays intrinsic to the bin-resolved larval scheme, although our model does not consider larval cannibalism, and thus could exaggerate the magnitude of this rebound effect.

Our model still does not consider other environmental factors such as photoperiods. Photoperiod is an important factor that triggers a diapause in *Ae. albopictus* in temperate regions [30]. The non-inclusion of photoperiods could explain simulated year-round activity of this mosquito in southern Italian cities, such as Palermo, where recent field observations tend to suggest a 10 months activity season [76]. However, modelling studies have highlighted that *Ae. albopictus* could become homodynamic in southern Mediterranean countries in the near future [20], a claim supported by the recently reported activity of *Ae. albopictus* during the 2022–2023 winter season in Attica (Greece), where it was found in large numbers [77]. Even though most winter observations in Italy, Albania and Spain are sporadic and in low numbers, *Ae. albopictus* has shown a remarkable degree of ecological plasticity in the past [78], with diapause adaptation to local climatic conditions [79,80]. There is thus a need to extend surveillance periods outside the usual expected activity range of *Ae. albopictus*.

We have modelled mosquito dynamics, but we did not consider pathogen transmission in our modelling framework. However, simulated hotspots coinciding with densely populated areas match reported autochthonous cases of chikungunya in Ravenna in 2007, and recently observed transmission of dengue virus in the Lazio region and in Lombardia in 2023. Future modelling efforts could focus on developing early warning tools based on numerical weather prediction systems as well as producing higher resolution risk estimates to guide control and surveillance activities.

## Data Availability

All data used in this study are open source and freely accessible from their respective citations. The model is open source and can be found, installed and used at the model's website https://users.ictp.it/~tompkins/vectri/. The model version 1.11.3, used in this study, has been archived in the Zenodo repository [81]. Instructions on how to reproduce our results, including the model inputs and post-processing files can be found at [82]. The ovitrap surveillance data used in this study was last accessed on the 2 February 2023 and is publicly available at https://zanzaratigreonline.it/it/monitoraggio/dati-di-monitoraggio. Supplementary material is available online [83].
